# Novel myosin-based therapies for congenital cardiac and skeletal myopathies

**DOI:** 10.1136/jmedgenet-2016-103881

**Published:** 2016-07-13

**Authors:** Julien Ochala, Yin-Biao Sun

**Affiliations:** 1 Centre of Human and Aerospace Physiological Sciences, King's College London, London, UK; 2 Randall Division of Cell and Molecular Biophysics, British Heart Foundation Centre of Research Excellence, King's College London, London, UK

**Keywords:** Congenital heart disease, Cardiomyopathy, Muscle disease

## Abstract

The dysfunction in a number of inherited cardiac and skeletal myopathies is primarily due to an altered ability of myofilaments to generate force and motion. Despite this crucial knowledge, there are, currently, no effective therapeutic interventions for these diseases. In this short review, we discuss recent findings giving strong evidence that genetically or pharmacologically modulating one of the myofilament proteins, myosin, could alleviate the muscle pathology. This should constitute a research and clinical priority.

In cardiac and skeletal muscles, the contractile machinery consists of assemblies of interdigitating myofilaments integrated in highly ordered entities termed sarcomeres.^1^ Mutations in genes encoding proteins located in the sarcomeres have been associated with various functional effects at the myofilament level grandly contributing to a wide range of clinical phenotypes such as hypertrophic cardiomyopathy (HCM), dilated cardiomyopathy (DCM), restrictive cardiomyopathy (RCM), nemaline myopathy or Laing early onset distal myopathy.^2^ ^3^ No treatment exists for these sarcomeric diseases. The current guidelines state that therapeutic interventions should simply target symptoms relief.^4^ The present short review article aims to briefly summarise the recent advances in our understanding of the molecular pathogenic mechanisms and the rationale for further testing myofilament-based therapeutic interventions involving the molecular motor, myosin.

## Sarcomeric mutations and clinical phenotypes

Here, the focus is on congenital cardiomyopathies and skeletal myopathies caused by dominant inherited and de novo mutations in genes encoding sarcomeric proteins. Mutations in MYH7 and MYBPC3 encoding for β-myosin heavy chain and myosin-binding protein C, respectively, are responsible for more than 70% of identified cardiac cases.^5^ Other genes such as MYL2, MYL3, ACTC1, TNNT2, TNNI3, TPM1, *TTN* and *DES* are also affected but account for less than 5% each.^5^ These gene defects lead to HCM, which is associated with left ventricular hypertrophy, small cavity size and vigorous systolic function.^6^ They can also induce DCM, RCM and left ventricular non-compaction cardiomyopathy, which are also characterised by enlarged left ventricles but with various effects on systolic function.^7–9^ Skeletal muscle-related mutations have also been identified in many genes including *NEB* and *ACTA1* encoding for nebulin and skeletal α-actin, respectively, as well as *TPM2*, *TPM3*, *MYH7* and *MYH2.*^10^ ^11^ These mutations often interfere with the normal limb, masticatory and respiratory muscle functioning resulting in atrophy and impairing mobility, feeding and breathing.^10^ ^11^ The following is a non-exhaustive list of the most common diseases: nemaline myopathy,^11^ Laing early onset distal myopathy,^12^ actin myopathy,^13^ intranuclear rod myopathy,^13^ rod-core disease,^13^ congenital fibre-type disproportion,^13^ hyaline body myopathy,^12^ myosin storage myopathy,^12^ inclusion body myopathy^12^ and myofibrillar myopathy.^14^ Interestingly, a few of the above mutations, especially in the *MYH7* gene, are associated with cardiac and skeletal symptoms as the encoded protein, β/slow-cardiac myosin is present in both cardiac and skeletal muscles.^12^

## Hypercontractile or hypocontractile myofilaments

All these proteins are essential for muscle contraction. Briefly, in the sarcomere, calcium ions activate the thin filaments by directly binding to troponin C, allowing the movement of tropomyosin molecules to uncover myosin-binding sites on actin monomers. Myosin then binds and pulls on the thin filaments, shortening the sarcomere. Myosin cycling is triggered by the hydrolysis of ATP into ADP and inorganic phosphate. The release of phosphate is crucial for the myosin head-bending and force generation. Subsequently, when calcium ions are removed from the cytoplasm, the sarcomere relaxes. Other sarcomeric proteins such as titin, the intermediate filaments and Z-disc proteins are also important for mechanosensing and maintaining the sarcomeric structure during contraction.^15^ ^16^ When sarcomeric mutations occur, the genotype-phenotype relationships become complex. Muscle dysfunction usually depends on the mutation location within the gene, the consequence at the protein level and the secondary disease-related protein post-translational modifications.^2^ ^3^ Mutant proteins or post-translational modifications of sarcomeric proteins in the presence of mutations alter troponin C activation, tropomyosin conformational changes, myosin enzymatic ATPase activity, ultimately altering the duty ratio, which is the fraction of myosin heads forming strong force-generating interactions with thin filaments at any moment. Increasing or decreasing the duty ratio modulates force production, shortening velocity, power output and energy cost. Since the power output is the product of force and velocity of muscle contraction, any modulation of force or/and velocity would lead to an alteration in power. These molecular alterations thus result in either myofilament hypercontractility or hypocontractility.^3^ ^17^ In the case of HCM and DCM mutations, these phenomena overall affect the cardiac output and can induce various abnormalities and remodelling in myocardial structure and function, ultimately provoking obstruction, heart failure and/or sudden death. For instance, M531R mutants in the motor domain of the human β/slow-cardiac myosin molecule have been shown to be stronger motors and have been suggested to interrupt myosin head putative interactions with other proteins (eg, myosin-binding protein C) resulting in hyperdynamic heart.^18^ In contrast to congenital cardiomyopathies where a vast number of mutations have hypercontractile consequences, most of skeletal myopathies-related mutations induce hypocontractility and overall weakness,^17^ ^19–21^ with only a few exceptions contributing to muscle stiffness and/or hypertonia.^22^ ^23^

## Rationale for myofilament-orientated therapies

The current cardiac therapeutic interventions using small molecules have mixed long-term improvements on muscle pathology/remodelling or clinical outcomes. One potential explanation is that these particular interventions do not directly target the molecular pathogenic causes of the muscle diseases, that is, myofilament dysfunction. Hence, developing new therapies that precisely correct for the contractile alteration may represent novel and efficacious approaches to preventing onset and progression or treating muscle pathology in the setting of inherited cardiac and skeletal myopathies.^24^ ^25^ As myofilament activation requires the calcium ions, a few positive inotropes have been developed and subdivided into two categories, that is, calcium-mobilising agents and calcium sensitisers.^26^ These drugs could potentially increase myofilament function.^26^ Calcium-mobilising compounds, such as amrinone, milrinone and vesnarinone, act as phosphodiesterase 3 (PDE3) inhibitors and favour an increase in the amount of intracellular calcium ions released from the sarcoplasmic reticulum. Similarly, some of the calcium sensitisers, including levosimendan, a drug used in patients with heart failure, have some PDE3 inhibitory activity, but directly bind to troponin C, decreasing the concentration of calcium ions required to trigger muscle contraction, increasing cardiac efficiency.^27^ This is notably the case of MCI-154, pimobendan, sulmazole and bepridil.^28–31^ The main advantage of this class of drugs over calcium-mobilising agents is the stimulation of the activity without increasing the cytosolic calcium concentration. However, all the above positive inotropes have been observed to aggravate the cardiac phenotypes and provoke arrhythmias, hypotension and mortality in a number of heart diseases such as HCM. A few other small molecules have specifically been developed for skeletal myopathies such as CK-2066206, CK-2127107 or tirasemtiv.^19^ ^32^ ^33^ They specifically bind to the fast isoform of troponin C that is only present in skeletal myofilaments. Their clinical efficacy remains uncertain as they drastically slow the rate of calcium ions dissociation from troponin C, impairing the relaxation process.^33^

## Myosin as a preferential target

**Figure 1:**
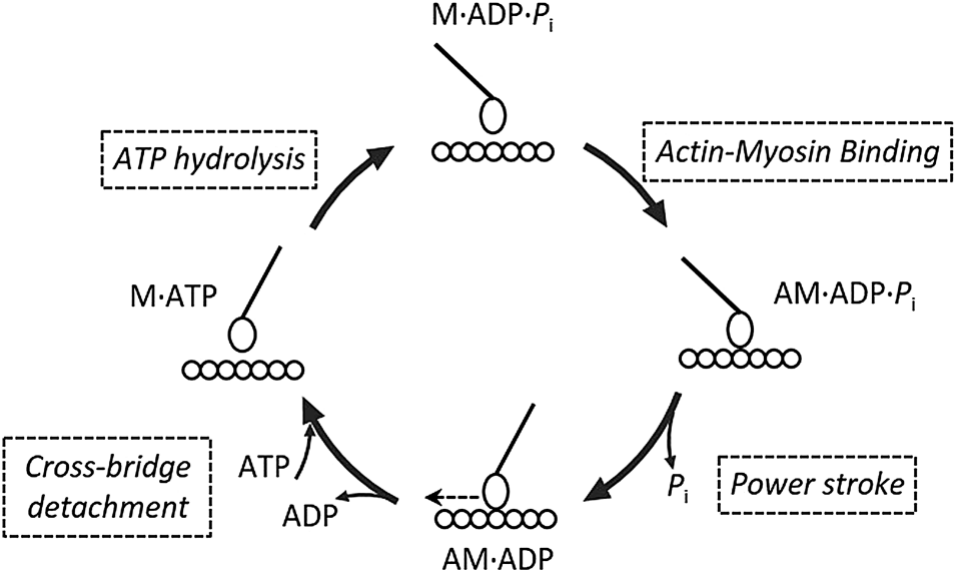
Schematic illustration showing actomyosin chemomechanical cycle. The transition from the weakly bound AM·ADP·*P*_i_ state to the strongly bound AM·ADP state is a key and rate-limiting step in the entire cycle that involves the release of P_i_ from the active site. Many myosin-base therapeutic or experimental interventions are targeting this step, for example, myosin activator (omecamtiv mecarbil) and inhibitors (MYK-461, blebbistatin, 2,3-butanedione monoxime, *N*-benzyl-*p*-toluene sulphonamide) in table 1. A, actin; AM, actomyosin complex; M, myosin.

In addition to troponin modulators, another recent experimental therapeutic approach consists of fine-tuning myosin function (table 1). The research compounds CGP-48506 and EMD-57033 directly affect myosin activity and the duty ratio^19^ ^34–36^ but have been observed to be toxic. Omecamtiv mecarbil, on the other hand, has a potential for therapeutic application.^37^ This strong positive inotropic small molecule binds directly to cardiac myosin and acts as an allosteric effector to enhance myosin motor activity and cardiac performance without increasing the intracellular calcium concentration.^37^ Omecamtiv mecarbil shifts the equilibrium of the myosin ATPase hydrolysis cycle towards products by accelerating phosphate release and leads to a faster transient from weak to strong actin-bound states^38^ (figure 1). Therefore, this drug exhibits the positive inotropic effects by increasing the duty ratio of myosin motor. In the heart, it prolongs the systolic ejection time without any increase in the rate of pressure development or myocardial oxygen demand. This shortens the diastolic filling time resulting in reduced coronary blood flow and risks of ischaemia. Clinical trials are encouraging and oral omecamtiv mecarbil could potentially be beneficial in the context of DCM and RCM where sarcomeric mutations reduce the myosin duty ratio. For HCM, negative inotropic agents are likely to have positive effects. Research molecules including blebbistatin and 2,3-butanedione monoxime have been developed and found to inhibit myosin activity by stabilising the myosin converter domain in a relaxed conformation, inducing a slowing of phosphate release and a decreasing in the duty ratio.^39^ ^40^ Unfortunately, the toxicity of these small compounds is quite high.^24^ Recently, a small molecule, MYK-461, which also interferes with the phosphate release step of the myosin ATPase cycle, has been shown the potential of therapeutic approach for HCM. MYK-461 inhibits cardiac myosin ATPase by slowing phosphate release and, consequently, reduces the myosin duty ratio and power output (figure 1). Tests on transgenic mice mimicking HCM and expressing particular mutations in the gene encoding for β-myosin heavy chain (leading to point substitutions: R403Q, R719W and R453C) demonstrate that MYK-461 reduces cardiac contractility and, when administered early in the course of the disease, it blunts the development of left ventricular hypertrophy.^41^ These phenomena are cardiac-specific and MYK-461 has no impact on skeletal muscle function.^41^

**Table 1:** Experimental approaches targeting myosin (derived from Ref. 24)

Approach	Compound	Target	Function
Pharmacological	Blebbistatin (research tool only)	Myosin class II All heavy chain isoforms	Inhibitor
	*N*-benzyl-*p*-toluene sulphonamide (research tool only)	Myosin class II Fast-skeletal heavy chain isoform	Inhibitor
	2,3-butanedione monoxime (research tool only)	Myosin class II All heavy chain isoforms	Inhibitor
	MYK-461 (preclinical—MyoKardia)	Myosin class II ß/slow-cardiac heavy chain isoform	Inhibitor
	Omecamtiv Mecarbil (clinical trial—Cytokinetics)	Myosin class II ß/slow-cardiac heavy chain isoform	Activator
	CGP48506 (preclinical—Novartis)	Myosin class II ß/slow-cardiac heavy chain isoform	Activator
	EMD57033 (preclinical—Merck)	Myosin class II ß/slow-cardiac heavy chain isoform	Activator
Genetic	*MYL4* incorporation (preclinical)	Myosin class II Atrial/fetal essential light chain isoform	Activator

Modulating the activation of myosin molecules in the context of skeletal myopathies can be achieved using gene therapy. Each myosin molecule is composed of two regulatory and two essential/alkali light chains.^42^ These latter are regulating the duty ratio,^42^ with some of the isoforms being very efficient force generators. This is specifically the case of the isoform encoded by the *MYL4* gene and only present in the heart and skeletal muscles from embryos.^42^ By implementing this isoform in the skeletal muscles of mice carrying one specific mutation in the *ACTA1* gene encoding for skeletal α-actin (H40Y replacement), myosin activity is enhanced and myofibres recover their force production, avoiding the development of muscle atrophy.^43^

## Conclusion

By alleviating muscle pathology, myosin activators and inhibitors represent promising new drug targets. Indeed, at the molecular level, these modify the ATPase activity, phosphate release, myosin-binding state and duty ratio, which are essential for regulating the force generation.
